# The Importance of Diaphragmatic Function in Neuromuscular Expression in Patients With Chronic Heart Failure

**DOI:** 10.7759/cureus.34629

**Published:** 2023-02-04

**Authors:** Bruno Bordoni, Allan R Escher

**Affiliations:** 1 Physical Medicine and Rehabilitation, Don Carlo Gnocchi Foundation, Milan, ITA; 2 Anesthesiology/Pain Medicine, H. Lee Moffitt Cancer Center and Research Institute, Tampa, USA

**Keywords:** aging, falls, fascia, myocardial infarction, chf, chronic heart failure, diaphragm

## Abstract

Chronic heart failure (CHF) is a set of symptoms and physical manifestations caused by the inability of the heart to perform its normal contractile function and satisfy the blood needs of all organs. This dysfunction leads to a non-physiological adaptation of all body systems, including the skeletal muscles and the diaphragm. The myopathy found in patients brings symptoms such as fatigue and intolerance to exercise, with an entity not always attributable to cardiac function. Neuromuscular incoordination is one of the symptoms related to CHF, causing an increased risk of mortality and hospitalization. The article reviews diaphragmatic adaptation in the presence of CHF and seeks to emphasize the importance of the diaphragm in understanding skeletal muscle incoordination in patients.

## Introduction and background

Myocardial infarction (MI) is the main presentation causing ischemic chronic heart failure (CHF) detection. In contrast, the most important causes leading to non-ischemic CHF are Myocardial infarction (MI), which is the main cause causing the due to metabolic dysfunction and hypertension [1]. The prevalence of CHF is continuously increasing, probably due to the parallel increase in aging and the clinical ability to deal with the disease [2]. The European Society of Cardiology (ESC) highlights two categories of patients based on the left ventricular ejection fraction (LVEF). ESC divides patients with reduced LVEF (41-49%) and patients with preserved LVEF (≥50%). Using the latter value, it is possible to identify above all female patients, older age, metabolic alterations, obesity, low values of natriuretic peptides, concentric remodeling, and minor disturbances of ventricular volumes [3]. A reduced percentage of LVEF highlights the presence of male patients, ischemic, eccentric remodeling, increased ventricular volumes and higher re-hospitalization, and more marked natriuretic peptide values [3]. There has been an approximately 10% increase in CHF with preserved LVEF over the last decade (women) and a higher prevalence of complications for markedly reduced LVEF (men), such as atrial fibrillation and decreased renal function [3]. Although the number of hospitalizations and the length of stay are not discriminating for the two phenotypes of patients identified by the ESC, the mortality rate is higher in people with a lower percentage of ventricular function [3]. The diagnosis of CHF is performed by echocardiography, electrocardiogram, and possible exercise test [3]. CHF involves not only the cardiovascular system (coronary artery disease, coronary microvascular dysfunction, hypertension, anemia), but since there is a picture of systemic inflammation (elevation of inflammatory markers, reduced lymphatic reserve), it negatively affects all body systems (diabetes, renal insufficiency) [3,4].

Osteoporosis is present in patients with cardiac dysfunction, with a high risk of bone fractures. A bone density decrease is an independent risk factor for developing CHF [5]. The overactivity of the sympathetic system could probably stimulate the bone production of alpha-adrenergic receptors and reduce the bone expression of beta2-adrenergic receptors, resulting in a consequent increase in osteoclast activity [5]. CHF alters the membrane permeability of the intestine, causing dysbiosis, probably due to a reduction in intestinal blood flow; there would seem to be an inverse relationship between the presence of zonulin in the blood (a protein derived from the intestinal mucosa) and ventricular function in patients [1]. Another non-physiological adaptation in the presence of CHF involves the musculoskeletal system, where we can find a picture of myopathy. The musculature of the patient with CHF undergoes a phenotypic change of the contractile fibers (from aerobic to anaerobic fibers), sarcopenia, atrophy of all types of fibers, apoptosis, fibrosis, and a decrease in expressed strength [6]. The decreased perfusion to the muscles and a widespread and active inflammatory system cause structural and morphological alterations of the skeletal muscle. The presence of metabolic dysfunctions (diabetes and obesity) can stimulate the autocrine and paracrine production of inflammatory muscle substances such as myokines (irisin, myostatin, type 15 and 6 interleukins, growth differential factor-11 and others), which can create a metabolic environment leading to myopathy before cardiac dysfunction is identified [6]. Elevated levels of pro-inflammatory myokines can induce pathological cardiac adaptations, creating a vicious circle [6]. Myopathic feedback is higher in patients with a low LVEF percentage than in patients with a preserved value; probably, the ubiquitin-proteasome-system (UPS) is more active than in subjects with a ventricle with preserved function [7]. Myopathy plays a vital role in explaining the patient's decreased tolerance to physical effort, which is not always related to cardiac contractile capacity [8,9]. The decreased physical work capacity and the pathological adaptation of the skeletal musculature lead to an increased risk of falls (and neuromuscular incoordination) [10]. A muscle not always considered in chronic pathologies is the diaphragm muscle, which adapts pathologically in the presence of CHF [9].

The article reviews the non-physiological adaptation of the main respiratory muscle, highlighting some functions related to the neuromotor behavior of the patient. This information can help understand the patients needs better.

## Review

Diaphragm adaptation in patients with CHF

**Figure 1:**
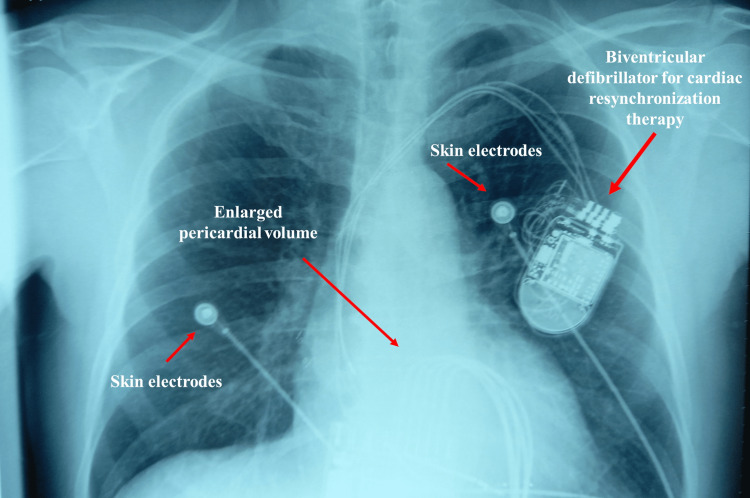
Patient with an implanted biventricular defibrillator for cardiac resynchronization therapy (PM CRT-D) with chronic heart failure (hypokinetic cardiomyopathy). The ejection fraction (EF) is 32%. Bordoni Bruno owns the figure.

After an ischemic heart attack, before chronic dysfunction occurs, the diaphragm muscle on an animal model undergoes an alteration of the transcript of the ribonucleic acid (RNA) sequencing [11,12]. This altered gene expression causes protein synthesis of some poorly functional contractile cell structures, such as the actomyosin complex, Z-band, and Z-disc proteins [11]. Other codings are altered, such as those responsible for the production of myokines (interleukin-6), metabolic cascades (g-protein-coupled receptor signaling pathway and protein kinase A catalytic subunit binding), and oxidative enzymatic behaviors (glutathione transferase activity) [11]. The neuromotor junction has dysfunctional genes, in particular, for structures that maintain stable the electrical relationship between the phrenic nerve and sarcolemma, such as muscle-specific tyrosine kinase (MuSK, transmembrane protein) and other proteins (dynactin-1, docking protein-7, kyphoscoliosis peptidase) [11]. After myocardial infarction, the patient suffers from fatigue and decreased inspiratory force [12]. These adaptations are emphasized in the evolution of ventricular dysfunction chronicity (and by aging). In an animal model, the presence of CHF presents alterations at the level of diaphragmatic deoxyribonucleic acid (DNA), in particular, for the structures responsible for aerobic metabolism, causing an increase in cellular oxidation [13]. In a human model, it increases intramuscular adipose tissue and connective tissue (a sign of atrophy), with a decrease in maximal inspiratory pressure (MIP) [14,15]. The diaphragmatic fibers have a decreased ability to release calcium, which slowness is reflected in a less effective contraction, with a decline in MIP of about 30% [15]. The amount of sarcomeric proteins, such as titin (shock absorber protein) and myosin heavy chain, is reduced; such adaptations will make the fiber frailer and have less expressed force capacity [15]. The literature does not seem univocal in the information concerning a phenotypic change of the contractile fibers on the human model; probably, elderly patients could have an increase in red-oxidative fibers and a decrease in aerobic-glycolytic ones [15]. The functional decrease of the diaphragm can be linked to various metabolic and endocrine causes. A constant low-grade inflammation stimulates the production of inflammatory cytokines (tumor necrosis factor-alpha - TNF-alpha, interleukin-6, interleukin-1beta); cytokines would stimulate the production of excess nitric oxide (iNOS) and a strongly oxidized cellular environment [16]. In particular, TNF-alpha stimulates myostatin synthesis and protein degradation [16]. Elevated levels of angiotensin type II (ANG2) in patients with CHF cause mitochondrial dysfunction by stimulating Nicotinamide adenine dinucleotide phosphate (NADPH) oxidase, which is the major source of reactive oxygen species [15]. NAD(P)H phosphorylates NADPH oxidase 2 (Nox2) and neutrophil cytosol factor 1 (p47phox), which are vital steps to activate myocyte oxidation [15]. Cytokines and ANG-2 can stimulate the enzyme sphingomyelinase in diaphragm muscle, accumulating ceramide and increasing protein dysfunction [15]. High circulating values of ANG2 prevent a correct synthesis of insulin-like growth factor 1 (IGF-1); the latter reduction leads to greater atrophy [16]. We find a reduced protein synthesis, stimulation of proteolysis, and an inflammatory environment. The result is a dysfunctional diaphragm (Figure 1).

The importance of the diaphragm muscle in patients with CHF in neuromotor expression

We know that patients with CHF suffer from neuromuscular incoordination. This condition leads to an increased risk of falls. Patients are approximately 43% more likely to fall than patients without CHF; muscle incoordination is a predictor of mortality and hospitalization [17]. Approximately 82% of patients have impaired gait, due to a lack of coordination, compared to subjects without CHF [17]. This motor dysfunction is found in NYHA class II and overweight or obese patients [17]. The diaphragm is hardly considered to understand this neuromotor situation. However, there is a tendency to explain this clinical phenomenon with the weakness of the limbs or an altered function of the nervous system [17]. Diaphragm dysfunction predicts physical activity intolerance in the patient, independently of ventricular dysfunction [18]. The function of the diaphragm is evaluated with instruments (X-ray, fluoroscopy, ultrasound, magnetic resonance, computerized axial tomography, trans diaphragmatic pressure, MIP, electromyography, airway occlusion pressure, sniff nasal inspiratory pressure) and through non-instrumental-and-non-invasive-tests (Bordoni diaphragmatic test) [18,19]. A close parallel exists between optimal diaphragm function and neuromotor strength/coordination [19]. Central pattern generator (CPG) is located between the midbrain, pons, and medulla and is responsible for managing breathing and non-respiratory actions related to the diaphragm [19]. We find different areas within the CPG, such as the pre-Bötzinger complex (preBötC), the caudal ventral group (VRGc), the rostral ventral group (VRGr), the parabrachial/Kölliker-Fuse complex and the nucleus of the solitary tract (NTS) of the vagus nerve [19]. When the diaphragm moves for inspiration, with a caudal and anterior vector, it stimulates multiple visceral (visceroceptors such as baroreceptors and others) and somatic receptors (proprioceptors, interoceptors, exteroceptors, and others); this receptor activation occurs thanks to the downward traction (felt above all by the thoracic area) and by the pressure generated in the abdominal area [20].

Approximately 90% of the collected information will be carried to the NTS, while approximately 10% will be carried toward the spinal trigeminal nucleus. NTS will send the received afferents to the cerebellum and vestibular area. The cerebellum and the vestibular area will send back various information to the NTS, which, with the new directives acquired, will send afferents toward the limbic area and the M1 motor cortex [19,20]. The limbic area (a periaqueductal gray area, pituitary, thalamus, and hypothalamus) will be critical for proper neuroendocrine response to required movement, while area M1 is crucial for spinal motor commands. The limbic area and M1 will send processed information back to the NTS. The latter will respond by sending descending afferents to the rostral ventrolateral medullary area, where the sympathetic system will receive inhibitory information [19,20]. Increased parasympathetic activity will improve the electromyographic spectrum, consequently increasing motor response coordination (greater strength, better balance, better posture) [19,20]. Each eupnoeic act will determine an optimal response to voluntary movements, such as walking, improving the ability to keep the body balanced. Patients with CHF have a diaphragm position with an inspiratory attitude, with further limitation of movement [21]. We can strongly hypothesize that a diaphragm dysfunction in patients with CHF is responsible for the neuromotor impairment, leading to the increase in falls and the percentage of mortality and hospitalization.

Future perspective

Considering that the non-physiological adaptation of the skeletal muscles and the diaphragm can be considered more important than the cardiac adaptation, the clinician should consider the patient's respiratory aspect [13]. There is the potential to improve the clinical picture, also passing through the diaphragm [18]. Respiratory muscle rehabilitation can improve diaphragm function, probably by changing metabolic and hormonal abnormalities [18]. Pulmonary rehabilitation improves contractile strength, the baroreceptor response quality, and the diaphragm's thickness; it decreases the systemic sympathetic response, lengthening the onset of contractile fatigue [15,18]. In the future, further studies should be focused on the diaphragm muscle, adding new knowledge bricks to understand the patient better.

## Conclusions

Chronic heart failure (CHF) is a growing pathology, both due to the increase in the age of the population (aging) and thanks to the improvement of the clinical, pharmaceutical, and instrumental approaches. Many pathological systemic adaptations affect the patient with CHF, such as respiratory, metabolic, bone, and other dysfunctions. Among the non-physiological adaptations, the diaphragm muscle undergoes harmful alterations that cause further complexity to the patient's clinical picture, such as fatigue and neuromuscular incoordination. Each eupnoeic act stimulates a parasympathetic response which determines a more coordinated voluntary movement of the limbs and trunk. Considering that the diaphragm in patients with CHF undergoes different pathological alterations, we can assume that the increase in the patient's neuromotor incoordination can be traced back to the main respiratory muscle. Greater emphasis should be given to the diaphragm to understand the patient's clinical picture better.
